# Mid-Infrared Mapping of Four-Layer Graphene Polytypes Using Near-Field Microscopy

**DOI:** 10.1021/acs.nanolett.3c02819

**Published:** 2023-11-26

**Authors:** Daniel Beitner, Shaked Amitay, Simon Salleh Atri, Andrew McEllistrim, Tom Coen, Vladimir I. Fal’ko, Shachar Richter, Moshe Ben Shalom, Haim Suchowski

**Affiliations:** † Department of Materials Science and Engineering Faculty of Engineering, Tel Aviv University Ramat Aviv, Tel Aviv 69998, Israel; ‡ University Centre for Nanoscience and Nanotechnology, Tel Aviv University Ramat Aviv, Tel Aviv 69998, Israel; § School of Physics and Astronomy, Faculty of Exact Sciences, Tel Aviv University, Tel Aviv 69978, Israel; ¶ National Graphene Institute, University of Manchester, Booth Street East, Manchester M13 9PL, United Kingdom; ∥ Department of Physics and Astronomy, University of Manchester, Oxford Road, Manchester, M13 9PL, United Kingdom

**Keywords:** Few-layer graphene, Raman spectroscopy, Mid-IR nano-imaging, Stacking order, Scanning near-field optical microscopy, Optical conductivity, Rhombohedral, Bernal

## Abstract

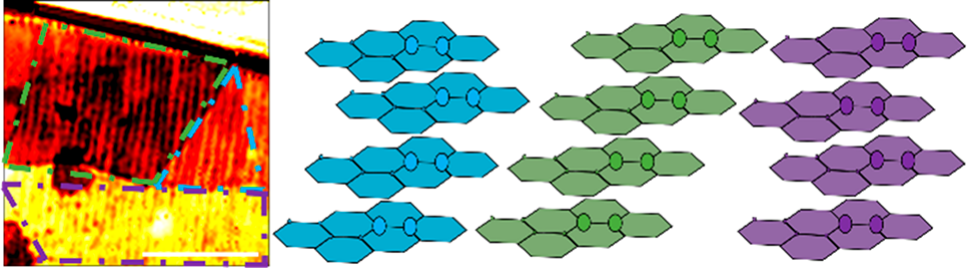

The mid-infrared (MIR) spectral region attracts attention for accurate chemical analysis using photonic devices. Few-layer graphene (FLG) polytypes are promising platforms, due to their broad absorption in this range and gate-tunable optical properties. Among these polytypes, the noncentrosymmetric ABCB/ACAB structure is particularly interesting, due to its intrinsic bandgap (8.8 meV) and internal polarization. In this study, we utilize scattering-scanning near-field microscopy to measure the optical response of all three tetralayer graphene polytypes in the 8.5–11.5 μm range. We employ a finite dipole model to compare these results to the calculated optical conductivity for each polytype obtained from a tight-binding model. Our findings reveal a significant discrepancy in the MIR optical conductivity response of graphene between the different polytypes than what the tight-binding model suggests. This observation implies an increased potential for utilizing the distinct tetralayer polytypes in photonic devices operating within the MIR range for chemical sensing and infrared imaging.

There is a growing interest in optically active materials in the mid-infrared (MIR) spectral region, particularly within the atmospheric window between 8.5 and 12 μm, due to the presence of vibrational absorption bands for numerous materials.^1,2^ Additionally, the vibrational excitation within this range exhibits a large extinction coefficient, enabling highly accurate chemical analysis.^3,4^ MIR photonic devices have found applications in various fields, including MIR broadband, ultrafast light sources,^5−8^ polarizers,^9^ thermal monitoring,^10^ and IR imaging.^11^ While the demand for MIR detection and imaging is evident, the search for materials that exhibit broad-band acceptance yet display selectivity in the MIR range is still in its infancy. Two-dimensional (2D) materials possess several properties that make them excellent candidates for MIR photonic applications, including modifiable bandgaps, small size, and high carrier mobility.^2^

Graphene, the first discovered 2D material, possesses several properties suitable for MIR devices, such as broadband absorption and the ability to modify plasmonic excitations through electrostatic gating.^12,13^ These properties can be further enriched by increasing the number of layers; for example, bilayer graphene is a semimetal where a band gap can be created with external displacement fields. Additionally, starting with three layers, the stacking order becomes a new degree of freedom, as recently shown with Bernal and rhombohedral stacks in trilayer graphene; here, the geometry of the unit cell determines a different band structure and, therefore, distinct properties for each polytype.^14^ Few-layer graphene (FLG) has demonstrated diverse properties, including quantum Hall states^15,16^ and superconductivity under an applied magnetic field for Bernal stacking.^17^ For rhombohedral stacking, multiple phases of electronic systems were observed, including various quantum Hall effect phases,^18^ and superconductivity and unique topological states have been observed.^19−21^ The optical response of FLG indicates that the energy of the potential bandgap decreases toward the MIR region as the number of layers increases.^22,23^ Moreover, this phenomenon is also influenced by the combinatorial stacking order of the thicker films.^24−30^

Specifically, in tetralayer graphene (4LG), there are three possible polytypes (Figure 1A): Bernal (ABAB), which is the most energetically favorable polytype^31^ among the three; rhombohedral (ABCA), which is less common;^22,32^ and the third, a metastable ABCB/ABAC polytype.^30,31^ Importantly, the ABCB/ACAB polytype is expected to have a bandgap of only 8.8 meV,^30^ and because its unit cell is noncentrosymmetric, it is the only one among them that generates a second harmonic generation (SHG) signal. While recent studies showed a different Raman and optical response (visible–near-infrared (Vis-NIR) range) of the three polytypes mentioned, their characterization in the MIR range is still lacking.

**Figure 1:**
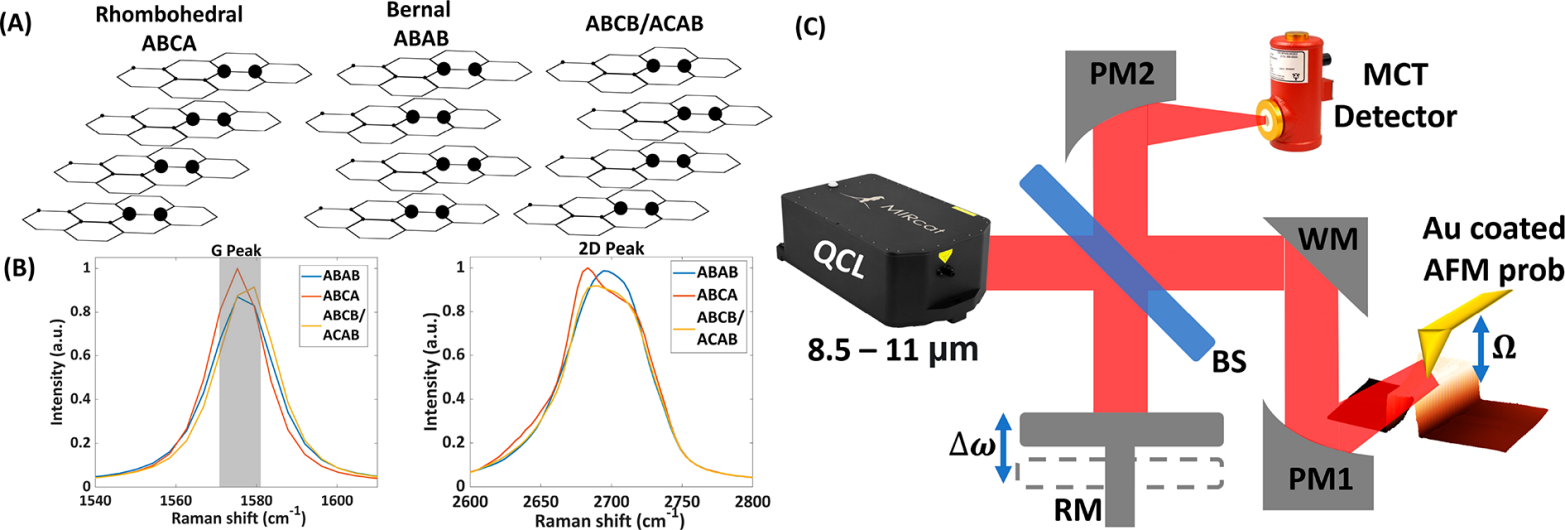
4LG far-field Raman identification and near-field setup. (A) Different possible polytypes of 4LG, which are characterized by shifted carbon pairs in each graphene layer. The ABCB/ACAB polytypes represent two possible stackings of the 4LG, which are identical when flipped. (B) Schematic of the s-NSOM system utilized in this work. The abbreviations BS, PM, WM, RM, and QCL correspond to the beam splitter, parabolic mirror, wedge mirror, reference mirror, and quantum cascade laser, respectively. (C) Raman peak measurements are obtained using a 532-nm laser source for the 2D and G peaks of each observed 4LG polytype. Subtle differences in peak shape can be observed, particularly for the ABAB and ABCA stackings.

In this study, we investigate and characterize the optical response of all polytypes of 4LG. We utilize far-field Raman and SHG imaging techniques in the NIR range to accomplish this. Additionally, we introduce the use of a scattering-scanning near-field optical microscopy (s-SNOM) in the MIR regime for the first time to study the different polytypes of 4LG. Our results demonstrate that the combination of 2D Raman and SHG analysis effectively detects the different polytypes of 4LG. However, the resolution limitations of far-field imaging restrict its ability to investigate the optical properties of ABCB/ACAB stacking, which is less stable compared to Bernal or Rhombohedral stacking and typically represents only a small fraction of the total flake area.^30−32^ By utilizing an s-SNOM, we overcome these limitations and can study even nanometer-sized ABCB/ACAB flakes. We also employ the s-SNOM technique to measure and analyze the near-field optical response at energies of 0.63 eV and 0.1–0.15 eV. While measurements at 0.63 eV yield similar results as previous works,^22,32^ we are the first to measure these compounds in the 0.1–0.15 eV range, which overlaps with the 8–11.5 μm MIR atmospheric window. Surprisingly, although theoretical spectral analysis does not predict any variations between polytypes along the MIR spectra, we observe that we can distinguish all different polytypes of 4LG. Through point spectroscopy and s-SNOM measurements, we obtain the optical response of each 4LG polytype across the 8.5–11 μm range. We compare our findings to theoretical results modeled using an expanded finite dipole model (FDM).^33^ The FDM model enables us to calculate the s-SNOM optical phase and amplitude for each 4LG polytype based on theoretical optical conductivity values obtained from a tight-binding model of 4LG. Our results highlight the strength of s-SNOM in the MIR region for identifying and characterizing other FLG polytypes. Moreover, they suggest the potential of utilizing 4LG as next-generation multicolor optical sensors and imaging devices.

FLG samples were prepared by mechanically exfoliating the flakes onto a SiO_2_/Si substrate (90 nm SiO_2_) using tape. 4LG flakes are identified by optical contrast and verified with atomic force microscopy (AFM) measurements. We initially opted to deposit the flakes on a SiO_2_/Si substrate to facilitate the necessary visible and Raman measurements. s-SNOM MIR measurements on SiO_2_ substrates are difficult due to noise from the SiO_2_ phonon in that range. However, due to the instability of ABCB/ABAC polytypes that undergo a transition to the more stable Rhombohedral or Brenel phases, during the transfer process, we have used a SiO_2_ substrate that allows us to measure all three polytypes.

Furthermore, the stacking configuration is identified by Raman microscopy performed using a commercial WITEC (Oxford Instruments) alpha300 Apyron confocal microscope equipped with a UHTS 600 mm focal length spectrometer. In Figure 1B, we present the measured 2D and G Raman peaks for each of the polytypes using a 532 nm laser source, which exhibits small differences in peak shape for each polytype. By leveraging these subtle variations between the Raman peaks, we mapped each polytype with a different contrast.^14,32^ Furthermore, second harmonic generation (SHG) microscopy was used to detect the noncentrosymmetric ABCB/ACAB polytype, by using a 1064-nm laser source.

The s-SNOM measurement system used in this study (Neaspec Attocube) is illustrated in Figure 1C. A parabolic mirror focuses a light source onto a conductive AFM probe (OPUS Model AC160GG, MikroMasch) operating in tapping mode. The system collects the backscattered light from the tip and directs it to an optical detector. To isolate the near-field signal from reflective far-field noise, a lock-in amplifier is employed to demodulate the signal to higher harmonics of the tapping frequency of the probe. A pseudoheterodyne measurement could be performed using a Michelson interferometer and a vibrating mirror, further reducing far-field effects. This configuration allows for recording both the optical amplitude and phase, denoted as σ_*n*_ = , where σ_*n*_ is the *n*th-order harmonic of the s-SNOM total signal, *S*_*n*_ the amplitude of the s-SNOM total signal, and ϕ_*n*_ the phase of the s-SNOM total signal.

The far-field and near-IR near-field optical measurements of the 4LG flake are shown in Figures 2 and 3. Figure 2A presents the optical image of the studied FLG flake. The central part of the flake is a 4LG surrounded on both sides by a five-layered region. The red region in Figure 2A was scanned using Raman microscopy (Figure 2B). Here, different contrasts within the 4LG part represent the three different polytypes. The map is constructed by integrating the Raman spectrum between 1572 cm^–1^ and 1582 cm^–1^ in each pixel (with background subtraction), and we discern between each polytype by the shape of the G peak. We further confirm the presence of the ABCB/ACAB polytype by mapping the SHG in the area, as is shown in Figure 2C, where the signal is present only in the triangular area of this polytype. (See more details in the Supporting Information.)

**Figure 2:**
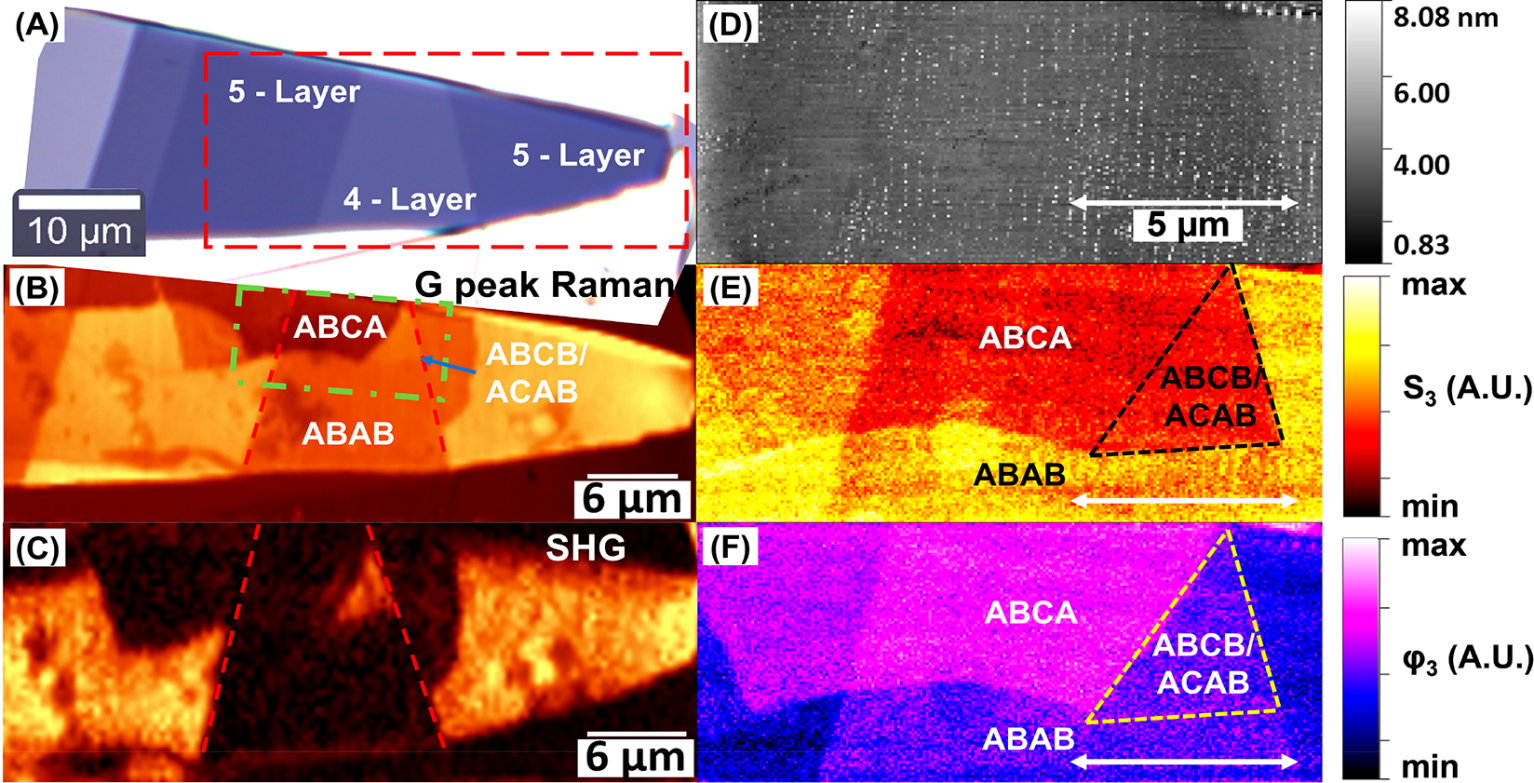
Far-field Raman and SHG measurements and 2 μm near-field optical scans on a tetralayer graphene (4LG) sample. (A) Optical microscopy image of the studied flake. The contrast of each section of the flake is related to the number of graphene layers in it. The flake has a 4-layer section between two 5-layer sections. (B) A G peak Raman scan of the flake was conducted, with two dashed red lines indicating the 4-layer section. The shaded area in Figure 1B indicates the filter used to generate the image. The scan shows two distinct peak shape sections, corresponding to ABCA and ABAB, and a third section not corresponding to either. (C) An SHG scan displays a small triangular zone with an SHG signal in the 4-layer section. (D) AFM topography scan of the 4-layer section of the flake, with boundaries marked by dashed red lines. (E, F) s-SNOM amplitude (panel (E)) and phase signals (panel (F)). The combination of amplitude and phase images clearly shows all three possible 4LG polytypes.

Figures 2D–F presents the near-field measurements at 0.63 eV (2 μm). These measurements provide a more-detailed image of the distinct 4LG polytypes. The AFM topography scan of the 4LG region (Figure 2D) did not reveal any noticeable differences between the different polytypes. Figures 2E and 2F respectively display the amplitude and phase signals obtained from the third-harmonic-order deconvolution, *S*_3_, of the s-SNOM signal. It is evident that while the amplitude image only shows two of the regions observed in the Raman scan, the phase image uncovers a third region in the ABCA orientation (indicated by a yellow triangle). This third region exhibits a distinct phase value, compared to the ABCA polytype, suggesting that it could correspond to the elusive third polytype, ABCB/ACAB. The measured phase and amplitude values for this third region are similar to those reported in previous works by Wirth et al.,^32^ further supporting the conclusion that this region represents the ABCB/ACAB polytype.

To further investigate the characteristics of the polytypes in the MIR region, we conducted point spectroscopy s-SNOM scans ranging from 8.5 μm to 11 μm with 0.25 μm increments. These scans were performed using a tunable MIR QCL (MIRcat-QT, DRS Daylight Solutions). The optical response of the polytypes was measured using the same AFM probe and scan parameters.

Figure 3A displays representative NSOM images of the amplitude and phase for the 4LG sample in the 8.5–10.95 μm range. Despite the lack of distinct optical features of graphene in the MIR range, the amplitude and phase scans in the 8.5–10.95 μm energy range clearly reveal all three different polytypes. Compared to the 2 μm scan, the MIR scans provide more-detailed information about the sample, including defects that are not visible in the far-field or 2 μm scans, due to the higher signal-to-noise ratio. Additionally, in the 9.5 μm range, the amplitude image flattens, possibly indicating an interaction between the sample and the SiO_2_ phonon in that range.

**Figure 3:**
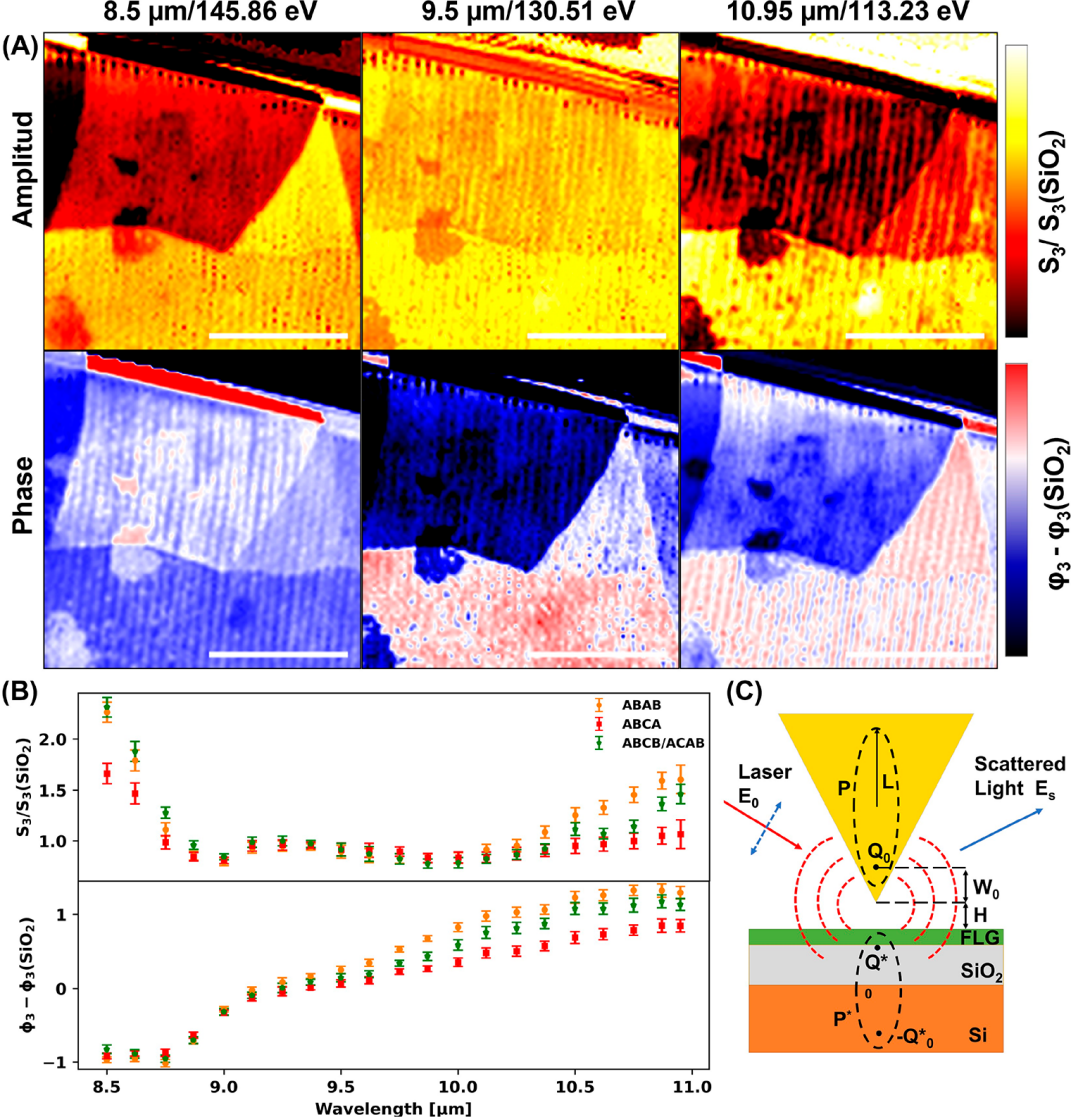
MIR 8.5–10.95 μm s-SNOM scan data for 4LG and a schematic of the analytical model used. (A) Amplitude (top) and optical phase (bottom) s-SNOM scan images of the 4LG sample. All images represent the third-harmonic-order deconvolution, *S*_3_, of the s-SNOM signal normalized by the substrate’s signal, which helps to reduce far-field noise and compensate for changes in laser power between scans. Horizontal lines visible in near-field results are due to previously performed SHG measurements of the sample. Each image color scale was set individually to maximize the contrast between the different polytypes in that image. All scale bars in the image are for 4 μm (B). The measured spectrum of the s-SNOM optical third harmonic amplitude and phase signal in the 8.5–11 μm range for each 4LG polytype. The spectrum shows a wavelength-dependent increase in the amplitude and phase differences between the different polytypes. (C) Schematic showing the FDM and its components. The incident beam (*E*_0_) generates a dipole *P* in the AFM probe (gold triangle). This dipole generates a mirror dipole P* through the interaction of the dipole charge *Q*_0_ with the surface. The interaction between the s-SNOM probe and the surface changes the scattered light (*E*_S_) going to the detector.

Figure 3B displays the measured spectra for each polytype of 4LG in the 8.5–10.95 μm range. To further analyze the data obtained from each scan, a Gaussian mixture model was employed to cluster the complex number representation of each pixel into similar clusters. This clustering method facilitates the efficient grouping of data from different regions of the scan that exhibit similar results. Furthermore, it allows us to combine scan data from both forward and backward scan directions (see more details in the Supporting Information), leading to improved data averaging.^34^ Using this technique, we can calculate the mean amplitude and phase for each of the different polytypes. To ensure a noise-free near-field signal, the data in this study were obtained from the third-harmonic-order deconvolution, *S*_3_, of the near-field signal and normalized by the signal from the SiO_2_/Si substrate (*S*_3_/*S*_3_ (SiO_2_/Si) and ϕ_3_–ϕ_3_ (SiO_2_/Si)).

The amplitude differences among the various 4LG polytypes also vary across this spectral range. At the shorter wavelengths of the MIR range, the near-field amplitudes of ABAB and ABCB/ACAB are nearly identical, while ABCA exhibits a lower amplitude. In the middle of the range, both ABCA and ABAB have similar amplitude values, but ABCB/ACAB experiences the lowest amplitude among the three. At longer wavelengths, there is a wavelength-dependent increase in the amplitude and phase differences among all three polytypes.

To compare the measured s-SNOM signal with the dielectric properties of the sample, we employ an expanded finite dipole model^35^ with an extension for multilayers.^36^ A schematic of this model is shown in Figure 3C, where the tip is represented as a spheroidal dipole, and the effective polarizability (α_eff_) of the tip–sample system can be calculated:
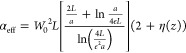
1
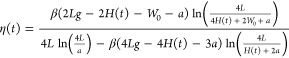
2where η is the near-field contrast factor, β the electrostatic reflection coefficient, *H* the tip–sample distance, *L* the effective spheroid length, *a* the tip radius, *g* the total induced charge on the AFM tip, and *W*_0_ ≈ 1.31*a*. As the AFM probe oscillates at frequency Ω, the tip–sample distance *H* is given as
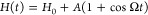
3where *H*_0_ is the minimum tip–sample distance and *A* is the tip amplitude. The field scattered from the AFM tip (*E*_sca_, representing the effective dipole) is proportional to
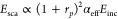
4where *E*_inc_ is the incoming field and *r*_*p*_ is the Fresnel reflection coefficient for *p*-polarized light. The near-field is extracted by taking the *n*th Fourier component of the scattered field:
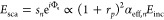
5

As the measured signal in the experiment is normalized against a reference signal (SiO_2_/Si), the results can be represented as
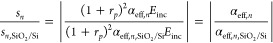
6and
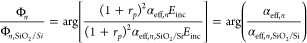
7

A comprehensive explanation of the model can be seen in refs (35 and 36). Model parameters (*L*, *A*, *a*, *g*) are adjusted using substrate data and compared to dielectric values for Si and SiO_2_ obtained from the literature. By employing the fitted model, we can calculate the expected amplitude and phase based on the theoretical dielectric values for the different polytypes of 4LG graphene (the fitting parameters used in the model can be found in the Supporting Information). The theoretical optical conductivity values for the various polytypes of 4LG were obtained from the tight-binding calculation conducted by McEllistrim et al.^37^ By fitting each point in our spectrum using our model, we can compare the theoretical s-SNOM results to our measurements.

Figure 4 shows a comparison of the model to the experimental results. The FDM employed in this study exhibited an excellent fit to the measured data within the lower range of the spectrum (8.5–9.5 μm), but its accuracy diminished at longer wavelengths. Notably, the model failed to explain the larger observed differences in the polytype phase and amplitude at longer wavelengths (10–11 μm). This discrepancy may be attributed to interactions between FLG and the SiO_2_ substrate. Previous studies have demonstrated that the interaction between graphene Dirac plasmons and the SiO_2_ substrate can influence the position and intensity of the SiO_2_ surface phonon, subsequently affecting the measured s-SNOM phase and amplitude for the system.^13,38−40^ Additionally, the ABCA polytype has been found to possess conductive surface states,^18,41^ which can interact with the SiO_2_ surface phonon, leading to further shifts in the s-SNOM results that are not currently accounted for in our model.

**Figure 4:**
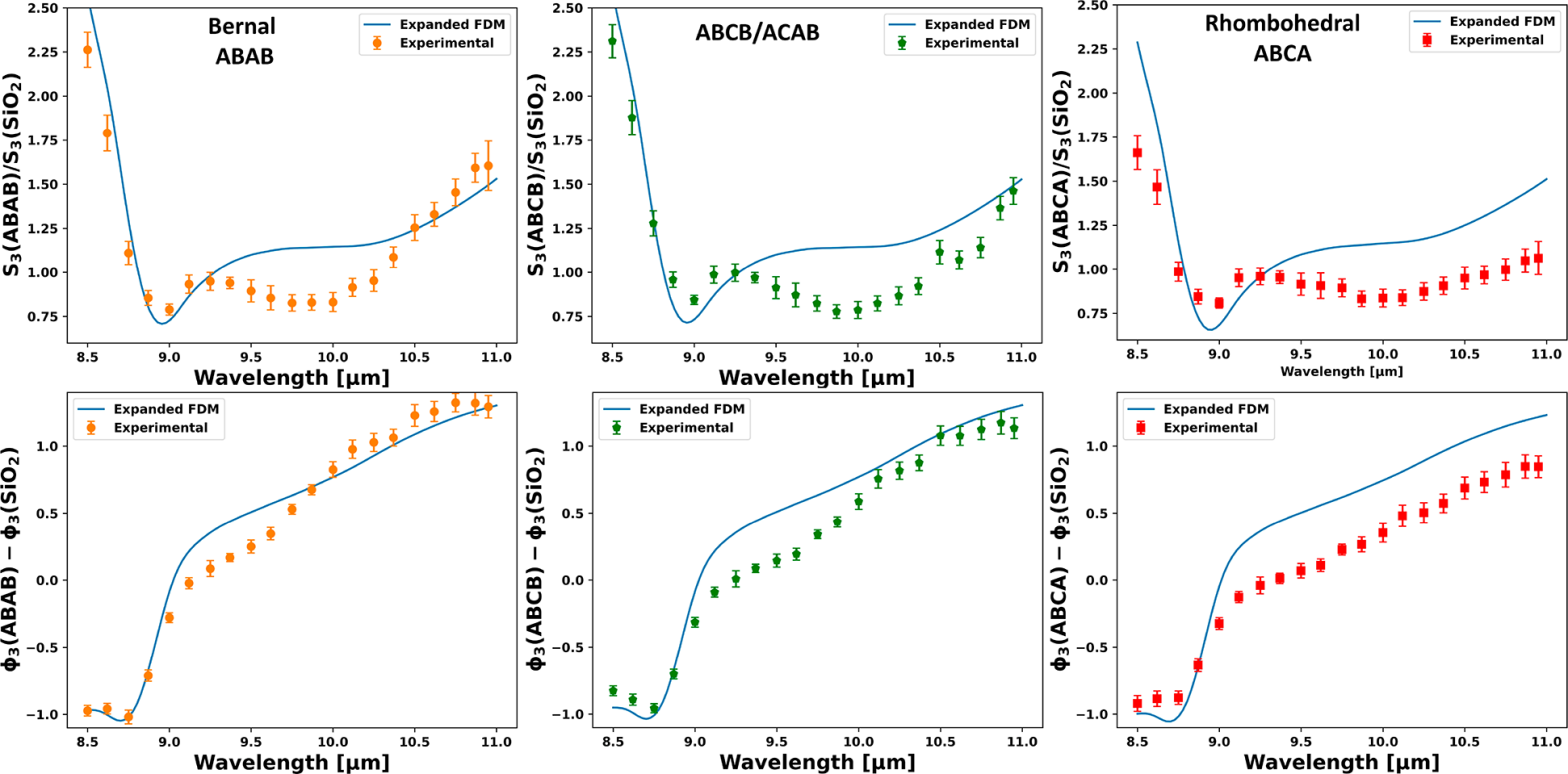
Point spectroscopy s-NSOM experimental measurements for 4LG graphene and the theoretical FDM model. The experimental results are compared with the predicted FDM model results for the optical conductivity calculated by the tight binding model.^37^ The FDM model can predict the lower part of the spectral scan range yet slightly deviates from the experimental measurements from 9.5 μm. Furthermore, experimental results show a larger amplitude and phase difference between the Bernal and rhombohedral polytypes than the theoretical in-plane optical conductivity predicted. (See the discussion in the text.)

To conclude, we unravel the optical response of the tetralayer graphene (4LG) polytypes in the mid-infrared (MIR) region. We have demonstrated the capability of s-SNOM to distinguish between different polytypes of 4LG, even in the absence of distinct optical features within that specific wavelength range. Notably, we have observed a significant dependence on wavelength in terms of amplitude and phase differences among the 4LG polytypes, particularly in the 10–11 μm region. This suggests that this spectral range holds the potential for identifying distinct polytypes of higher-order graphene stacking structures. The FDM model provides a good prediction for the amplitude and phase of the ABAB and ABCB/ACAB stackings, particularly within the 8.5–10 μm range. However, it does not fully capture the larger differences observed in ABCA stacking. This finding highlights the substantial material contrast and lack of optical features for 4LG graphene polytypes in the MIR range, which can be leveraged for MIR optical detectors and devices.

## Supporting Information Available

Analysis of Raman and SHG data to detect different 4LG polytypes; point spectroscopy data analysis method using the Gaussian mixture model sorting algorithm for fast near-field amplitude and phase extraction; detailed explanation of the calculation process for Fresnel reflection coefficient for use in finite dipole model, and the fitting parameters for the model used in this work (PDF)
