# Supplementary material for: Mid-Infrared Mapping of Four-Layer Graphene Polytypes Using Near-Field Microscopy
Source: Nano Lett. 2023 Nov 26;23(23):10758–64. doi: 10.1021/acs.nanolett.3c02819 (PMC10722527; doi:10.1021/acs.nanolett.3c02819)
Supplement: Supplementary file 1 — nl3c02819_si_001.pdf [file nl3c02819_si_001.pdf]

Mid Infrared Mapping of Four-Layer Graphene Polytypes using Near-Field Microscopy

Daniel Beitner,[*,†,‡,¶] Shaked Amitay,[¶] Simon Salleh Atri,[¶] Andrew McEllistrim,[§,‖] Tom Coen,[¶] Vladimir I. Fal'ko,[§,‖] Shachar Richter,[†,‡] Moshe Ben Shalom,[‡,¶] and Haim Suchowski[‡,¶]

†*Department of Materials Science and Engineering Faculty of Engineering, Tel Aviv University Ramat Aviv, Tel Aviv 69998, Israel*

‡*University Centre for Nanoscience and Nanotechnology Tel Aviv University Ramat Aviv, Tel Aviv 69998, Israel*

¶*School of Physics and Astronomy, Faculty of Exact Sciences, Tel Aviv University, Tel Aviv 69978, Israel*

§*National Graphene Institute Booth Street East, Manchester M13 9PL, UK*

‖*Department of Physics and Astronomy Oxford Road, Manchester, M13 9PL, UK*

E-mail: Beitner@mail.tau.ac.il

S1 Point spectroscopy data analysis

Raman and second harmonic generation (SHG) measurements were conducted using a commercial WITEC alpha300 Apyron confocal microscope. The microscope was equipped with a UHTS 300 mm focal length spectrometer and a 300 lines/mm grating. For Raman analysis, a 532 nm laser was employed, focused to a spot size of approximately 300 nm. The

raster scans were obtained with a step size of 250 nm, utilizing an average power of around 4 mW. Integration times were kept shorter than 1 second to prevent laser-induced heating.

To create Raman maps, photon counts were integrated within a specific range that optimized contrast between polytypes. Moreover, background subtraction was performed on the integral. As an example, in Figure 2B map, the range is displayed in Figure 1B. In this case, the integral yielded a high value for the rhombohedral polytype and a low value for Bernal. These findings align with established results [1–3]. Additionally, the appearance of ABCB was validated using the G-peak and M-peak (Figure S1).

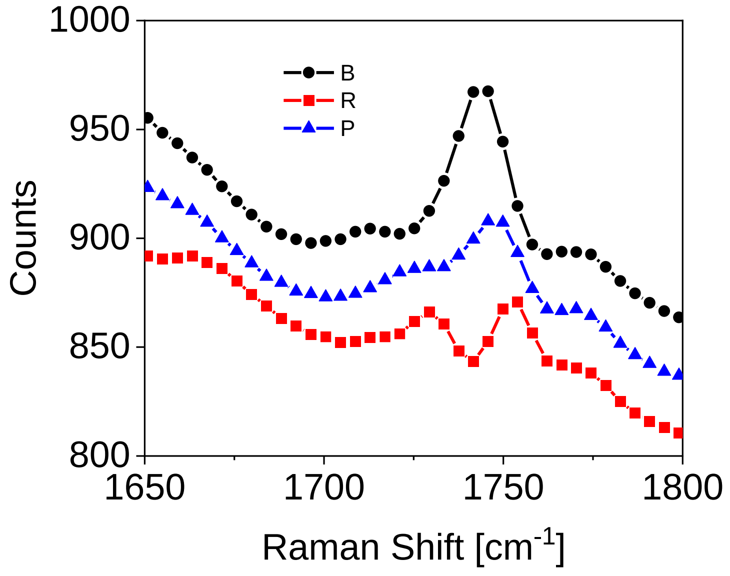

Figure S1: Raman spectra intensity for the M peak (≈1740 cm $^{-1}$) in tetralayer graphene. The spectra show the known shapes[3] concerning each polytype. ABAB/ABCA/ABCB are shown respectively with Black/Red/Blue lines.

The 1064 nm pulsed laser (15 ps, 50 MHz) is employed for second harmonic generation (SHG) in our study. It should be noted that only the non-centrosymmetric polytype, ABCB, can produce a signal, as ABAB and ABCB possess a center of inversion. Fig S2 showcases

spectra for each polytype, and it is demonstrated that only ABCB exhibits a signal at 532 nm. These findings align with previously documented results in trilayer graphene.[4]

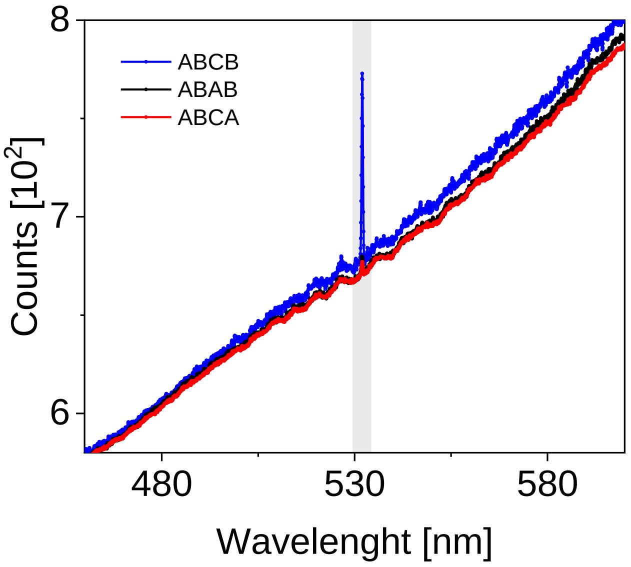

Figure S2: SHG intensity in tetra layer graphene. The spectra show a non-linear response (peak at 532 nm) only for the ABCB polytype (black line). No response is observed for the ABAB/ABCA (Black/Red) case.

S2 Point spectroscopy data analysis

In order to obtain spectral data from s-SNOM scans of our samples, we conducted a point spectroscopy experiment. This involved performing consecutive single-wavelength measurements under the same conditions using an AC160-GG AFM probe (OPUS) with a laser power of 3 mW. Figure S1A and B display the amplitude and phase results of a representative point spectroscopy spectral data point.

To analyze the resulting scan data, we employed a Python machine learning algorithm.[5] The analysis script utilized a Gaussian mixture model sorting algorithm[6] to categorize the scan into different sections of the sample area, such as Substrate, Graphite, ABAB, ABCB, ABCA, and defects. The Gaussian mixture model algorithm is an unsupervised machine learning technique designed to identify clusters of points in a dataset that share similarities.

It achieves this by using a probability distribution as a weighted sum of multiple Gaussian distributions for each potential cluster.

To facilitate the analysis, we transformed the amplitude and phase of each pixel in the scan dataset into complex numbers, represented as $S_n = \sigma_n e^{i\phi_n}$. This ensures that both phase and amplitude are on the same scale. Figure S1C demonstrates the complex representation of the data using a Red-Green color scale. This combined visualization of amplitude and phase contrast provides valuable insights into the scan data.

The dataset, in its complex number form, can be combined for both the forward and backward scan directions. This allows us to represent the data as a scatter plot in the complex plane. By employing the Gaussian mixture model algorithm,[6] we can automatically sort the different pixels into representative data clusters based on their similarities, measured by the probability Gaussian. Figure S1D displays the results of the Gaussian mixture model sorting on the image, where the color of each pixel corresponds to its assigned cluster. The excellent match between the assigned clusters and the image demonstrates the effectiveness of the sorting algorithm.

In our study, we extracted the s-SNOM amplitude and phase from the cluster centers, which represent the mean values of all data points belonging to each cluster. This approach allowed us to obtain an average measurement for each polytype region in both the forward and backward scan directions, while excluding data points corresponding to defects or contaminants on the sample surface.

S3 Extended Finite Dipole Model

The theoretical calculations of s-SNOM amplitude and phase in this paper were performed using a Finite Dipole Model (FDM)[7] with an extension for multilayers.[8] In the model, the sample is represented by the electrostatic reflection coefficient β in the formula for the

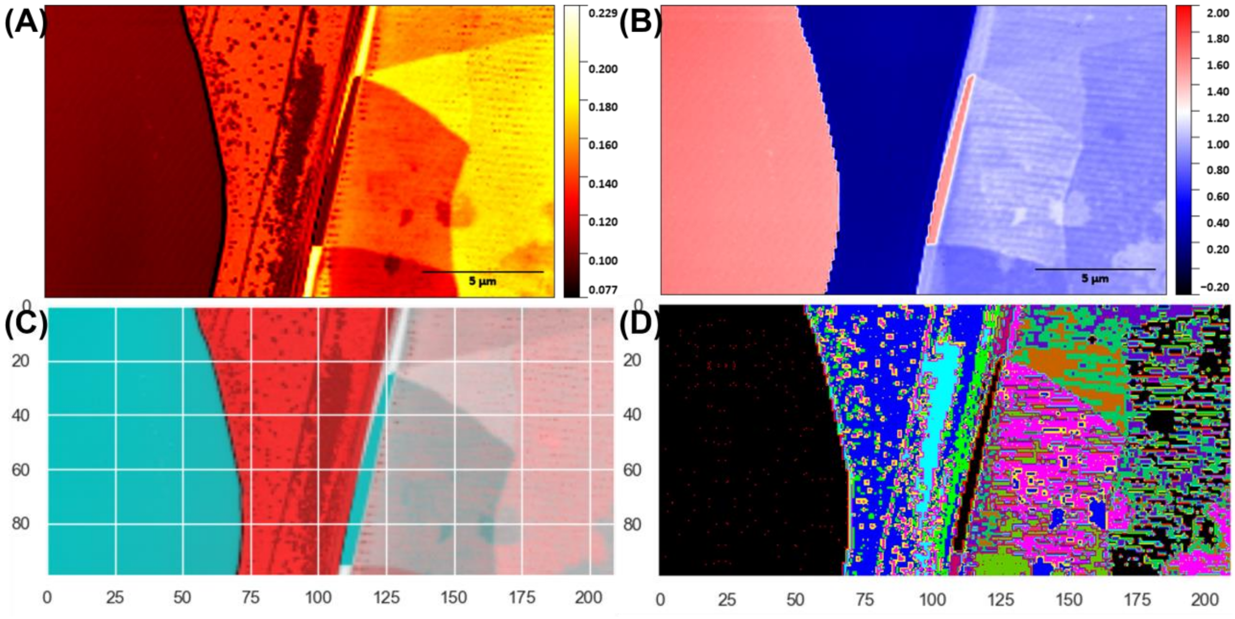

Figure S3: Data analysis of s-NSOM data at 10.87 microns. (A) - Amplitude results of the 4LG flake, highlighting the substrate, graphite, and flake. (B) - Phase results of 4LG flake. (C) - a complex color scan representation is presented, where real values are displayed on the red color scale and imaginary values on the green color scale. This combined visualization provides a comprehensive view of both amplitude and phase contrast in a single image. (D) - showcases the result of the clustering algorithm, with each pixel in the scan colored according to its assigned cluster identity. This clustering process allows for the identification and categorization of different regions or features within the scan based on their similarity.

polarizability of the AFM probe system (equation 2). For a layered system in the MIR, the electrostatic reflection parameter β can be replaced by the Fresnel reflection coefficient[8] at a representative dominant parallel wavevector $k_\parallel$:

$$\beta \approx r_p(k_\parallel, \varepsilon_i) \qquad \text{(S1)}$$

The Fresnel coefficient of reflection was calculated using Matrix Transfer Method (TMM)[9] for p-polarized light. The magnetic field at the interface of two materials (z=0) can be written as:

$$H_{1y} = \left(a_1 e^{ik_{1z}z} + b_1 e^{-ik_{1z}z}\right) e^{iqx}, \qquad z < 0, \qquad \text{(S2)}$$

$$H_{2y} = \left(a_2 e^{ik_{2z}z} + b_2 e^{-ik_{2z}z}\right) e^{iqx}, \qquad z > 0, \qquad \text{(S3)}$$

Where a_i and b_i are the coefficients of the magnetic field, k_{iz} is the out-of-plane component f the wavevector and q is the inplane component of the wavevector so that $k_i = \sqrt{\varepsilon_i}\omega/c = \sqrt{k_{iz}^2 + q^2}$ where ε_i is the dielectric permittivity in the respective layer, ω is the angular frequency and c is the speed of light in a vacuum. With the boundary conditions, we can find the following relation between the coefficient above the layer (a_1,b_1) and below the interface (a_2,b_2):

$$\binom{a_1}{b_1} = D_{12}\binom{a_2}{b_2} \quad where \quad D_{12} = \frac{1}{2}\begin{pmatrix} 1 + \chi + \xi & 1 - \chi - \xi \\ 1 - \chi + \xi & 1 + \chi - \xi \end{pmatrix} \tag{S4}$$

D_{12} is the interface transfer matrix for p-polarized light. The parameters:

$$\chi = \frac{\varepsilon_1 k_{2z}}{\varepsilon_2 k_{1z}}, \quad \xi = \frac{\sigma k_{2z}}{\varepsilon_0 \varepsilon_2 \omega} \tag{S5}$$

Where σ is the in-plane optical conductivity in the interface layer, which can describe the graphene layer. The propagation of light through a homogenous layer can be described by a propagation matrix over a distance Δd:

$$P(\Delta d) = \begin{pmatrix} e^{-ik_z\Delta d} & 0 \\ 0 & e^{ik_z\Delta d} \end{pmatrix} \tag{S6}$$

The TMM allows us to model the reflection coefficient for an arbitrary layered structure by the totaled transfer matrix M:

$$\binom{a_1}{b_1} = M\binom{a_N}{b_N}, \quad M = D_{12}P(\Delta d_2)D_{23}P(\Delta d_3) \dots P(\Delta d_{N-1})D_{N-1,N} \tag{S7}$$

And the reflection coefficient r_p:

$$r_p = \frac{M_{21}}{M_{11}} \tag{S8}$$

Our fitting found the best fit for $k_{\parallel}$ to be $1.7\times10^5 cm^{-1}$, which is similar to values reported in other works.[3,10] In our experiments, the substrate is coated with 90 nm of SiO_2. Therefore, the layered structure we modeled was air/Graphene/SiO_2/Si. The dielectric properties of

SiO_2 and Si were obtained from previous works,[12,12] and the optical properties for the tetralayer graphene polytypes were taken from a tight-binding model.[13] The fitting parameters were obtained by fitting the FDM to the results from scanning sections of the SiO_2/Si substrate without graphene. The fitting parameters used in this work are presented in Table S1:

Table S1: Parmeters used in FDM modeling of s-NSOM signal

Parameter	Value
Demodulation order n	3
Tapping Amplitude H	60 nm
Prob radius a	10 nm
Probe length L	100 nm
g	0.88+0.21i
SiO_2 thickness	90 nm

References

(1) Lui, C. H.; Li, Z.; Chen, Z.; Klimov, P. V.; Brus, L. E.; Heinz, T. F. Imaging Stacking Order in Few-Layer Graphene. Nano Lett 2011, 11 (1), 164–169. https://doi.org/10.1021/NL1032827/SUPPL_FILE/NL1032827_SI_001.PDF.

(2) Cong, C.; Yu, T.; Sato, K.; Shang, J.; Saito, R.; Dresselhaus, G. F.; Dresselhaus, M. S. Raman Characterization of ABA- and ABC-Stacked Trilayer Graphene. ACS Nano 2011, 5 (11), 8760–8768. https://doi.org/10.1021/NN203472F/SUPPL_FILE/NN203472F_SI_001.PDF.

(3) Wirth, K. G.; Hauck, J. B.; Rothstein, A.; Kyoseva, H.; Siebenkotten, D.; Conrads, L.; Klebl, L.; Fischer, A.; Beschoten, B.; Stampfer, C.; Kennes, D. M.; Waldecker, L.; Taubner, T. Experimental Observation of ABCB Stacked Tetralayer Graphene. ACS Nano 2022, 16 (10), 16617–16623. https://doi.org/10.1021/ACSNANO.2C06053/ASSET/IMAGES/LARGE/NN2C06053_0004.JPEG.

(4) Shan, Y.; Li, Y.; Huang, D.; Tong, Q.; Yao, W.; Liu, W. T.; Wu, S. Stacking Symmetry Governed Second Harmonic Generation in Graphene Trilayers. Sci Adv 2018, 4 (6). https://doi.org/10.1126/SCIADV.AAT0074/SUPPL_FILE/AAT0074_SM.PDF.

(5) Beitner, D.; Carmeli, I.; Zalevsky, Z.; Richter, S.; Suchowski, H. Coupled Molecular Emitters in Superstructures Interact with Plasmonic Nanoparticles. *Advanced Photonics Research* **2022**, *3*, 2100334.

(6) Pedregosa, F. et al. Scikit-learn: Machine Learning in Python. *Journal of Machine Learning Research* **2011**, *12*, 2825–2830.

(7) Cvitkovic, A.; Ocelic, N.; Hillenbrand, R. Analytical model for quantitative prediction of material contrasts in scattering-type near-field optical microscopy. *Optics Express* **2007**, *15*, 8550.

(8) Hauer, B.; Engelhardt, A. P.; Taubner, T. Quasi-analytical model for scattering infrared near-field microscopy on layered systems. *Optics Express* **2012**, *20*, 13173.

(9) Zhan, T.; Shi, X.; Dai, Y.; Liu, X.; Zi, J. Transfer matrix method for optics in graphene layers. *Journal of Physics: Condensed Matter* **2013**, *25*, 215301.

(10) Luo, W.; Boselli, M.; Poumirol, J.-M.; Ardizzone, I.; Teyssier, J.; van Der Marel, D.; Gariglio, S.; Triscone, J.-M.; Kuzmenko, A. B. High sensitivity variable-temperature infrared nanoscopy of conducting oxide interfaces. *Nature communications* **2019**, *10*, 2774.

(11) Shkondin, E.; Takayama, O.; Panah, M. A.; Liu, P.; Larsen, P. V.; Mar, M. D.; Jensen, F.; Lavrinenko, A. Large-scale high aspect ratio Al-doped ZnO nanopillars arrays as anisotropic metamaterials. *Optical Materials Express* **2017**, *7*, 1606–1627.

(12) Kischkat, J.; Peters, S.; Gruska, B.; Semtsiv, M.; Chashnikova, M.; Klinkmu¨ller, M.; Fedosenko, O.; Machulik, S.; Aleksandrova, A.; Monastyrskyi, G., et al. Mid-infrared optical properties of thin films of aluminum oxide, titanium dioxide, silicon dioxide, aluminum nitride, and silicon nitride. *Applied optics* **2012**, *51*, 6789–6798.

(13) McEllistrim, A.; Garcia-Ruiz, A.; Goodwin, Z. A.; Falko, V. I. Spectroscopic signatures of tetralayer graphene polytypes. *arXiv preprint arXiv:2302.07374* **2023**,
